# Modulations of genes related to gut integrity, apoptosis, and immunity underlie the beneficial effects of *Bacillus amyloliquefaciens* CECT 5940 in broilers fed diets with different protein levels in a necrotic enteritis challenge model

**DOI:** 10.1186/s40104-020-00508-4

**Published:** 2020-10-16

**Authors:** Kosar Gharib-Naseri, Juliano Cesar de Paula Dorigam, Kiran Doranalli, Sarbast Kheravii, Robert A. Swick, Mingan Choct, Shu-Biao Wu

**Affiliations:** 1 School of Environmental and Rural Science, University of New England, Armidale, NSW 2351 Australia; 2 Evonik Nutrition & Care GmbH, 10-B226, Rodenbacher Chaussee 4, 63457 Hanau-Wolfgang, Germany; 3 University of New England, Armidale, NSW 2351 Australia

**Keywords:** *Bacillus amyloliquefaciens*, Broiler, *Clostridium perfringens*, Gene expression, Necrotic enteritis, Probiotic

## Abstract

**Background:**

The ban of in-feed antimicrobial additives has negatively affected the poultry industry by causing necrotic enteritis (NE) to emerge in the flocks. Alternatives such as *Bacillus* probiotics have shown to be effective on eliminating the negative effects of this disease. Two experiments were conducted to investigate the effect of *Bacillus amyloliquefaciens* CECT 5940 (BA) in broiler chickens under NE challenge and/or fed diets with different protein levels.

**Methods:**

In both experiments, 480 day-old mix-sexed Ross-308 broilers were arranged in a 2 × 2 factorial arrangement of treatments. In experiment 1, the factors were NE challenge (yes or no) and probiotic (yes or no). In experiment 2, the factors were dietary crude protein levels (standard or reduced) and probiotic (yes or no) and were used under NE challenge condition. Oral administration of *Eimeria* oocysts (day 9) followed by inoculation with *Clostridium perfringens* (day 14 and 15) was used to induce NE challenge. On day 16, two birds from each treatment were gavaged with fluorescein isothiocyanate-dextran (FITC-d) and blood samples were collected for gut integrity evaluation, and jejunal samples were collected for gene expression assay.

**Results:**

In experiment 1, BA supplementation decreased caspase-3 (*CASP3*) (*P* < 0.001) and caspase-8 (*CASP8*) (*P* < 0.05) and increased occludin (*OCLD*) (*P* < 0.05) expression regardless of the challenge. Additionally, BA supplementation downregulated interfron-γ (*IFN-γ*) expression (*P* < 0.01) and upregulated immunoglobulin-G (*IgG*) (*P* < 0.01) and immunoglobulin-M (*IgM*) (*P* < 0.05) only in challenged birds. In experiment 2, the expression of genes encoding mucin-2 (*MUC2*) (*P* < 0.001), tight junction protein-1 (*TJP1*) (*P* < 0.05) and *OCLD* (*P* < 0.05) were upregulated by the addition of BA in the diet, regardless of the crude protein level. Further, BA supplementation downregulated *INF-γ* (*P* < 0.01) and upregulated immunoglobulin-A (*IgA*) (*P* < 0.05), *IgM* (*P* < 0.05) and *IgG* (*P* < 0.01) regardless of the crude protein level.

**Conclusion:**

These findings suggest that supplementation of BA in broiler diets can improve gut health by modulation of genes related to the mucosal barrier, tight junction, and immunity in broilers challenged by unfavourable conditions such as NE challenge.

## Background

The primary cause of necrotic enteritis (NE) in chickens is the bacterium *Clostridium perfringens.* The acute clinical form of this disease leads to significant flock mortality, whereas the subclinical form can decrease weight gain with a subsequent loss of productivity. The NE infection has become a significant economical concern for poultry farmers, especially in the subclinical form [1], leading to a US$6 billion annual loss to the global poultry industry [2]. In-feed antimicrobials show effective NE control, however, the ban of these additives has negatively affected the poultry industry by causing NE to emerge in the flocks [3]. Many alternatives to in-feed antibiotics have been explored to improve microbial balance and maintain growth performance of broilers [4]. *Bacillus* species are used as probiotics in animal industry and have shown many positive effects on maintained broiler health [5]. Because of the inherent capacity of *Bacillus* bacterium to withstand harsh conditions in feed processing such as pelleting and conditioning at high temperature and under harsh acidic conditions in the gut, they are a suitable option to be used in broiler diets. Among the *Bacillus* species, *B. amyloliquefaciens* (BA) has shown substantial antimicrobial activities [6, 7] and produces several extracellular enzymes such as α-amylase, cellulase, and proteases that enhance digestibility and absorption of nutrients [8]. These strains can also produce different bacteriocins, such as barnase and binase which can be effective in controlling *C. perfringens* [9, 10]. Furthermore, the quorum quenching activity of BA can disturb the quorum sensing signaling molecules of some pathogenic bacteria, which is essential for the communication and growth of these microorganisms, and thereby inhibit the growth of the harmful groups [11, 12].

The importance of the intestinal tract and its critical role in nutrient absorption and immune responses is evident [13]. The mucosal barrier mechanism in the small intestine serves as the body’s first line of defence and can maintain an essential barrier to microbial invasion and protect the intestinal epithelial cells [14]. Mucin proteins such as, mucin-2 (MUC2) and mucin-5 ac (MUC5ac, maintain a suitable thickness of the mucous layer, as this layer is often sloughed off by intestinal movements or microbial-derived factors [15]. Immunoglobulin proteins such as immunoglobulin-A (IgA), immunoglobulin-M (IgM) and immunoglobulin-G (IgG/ IgY) are present in the enterocyte brush border and are delivered to the mucus layer to perform immune exclusion and clearance of antigens [16]. Furthermore, intestinal absorbing epithelial cells are strongly connected by tight junction proteins such as claudin-1 (CLDN1), occludin (OCLD) and tight junction protein-1 (TJP1). The function of these proteins is necessary for controlling permeability on the paracellular pathways. Furthermore, while apoptosis (cell death) usually happens during development and aging, it can also occur in defense mechanisms such as DNA damage caused by UV light, ionizing radiation, disease or toxic agents [17]. Activation of caspase proteases correlates with the onset of apoptosis and cell death [18]. Sucrase-isomaltase (SI), ATPase Na^+^/K^+^ transporting subunit alpha-1 (ATP1A1) and glucose transporter-2 (GLUT2) proteins in the intestinal epithelium are closely associated with intestinal nutrient digestion and absorption capacity [19–21]. Altogether, the level of expression of these genes in the different groups of this study can illustrate a better understanding of BA supplementation in the birds.

The high cost of protein sources and environmental concerns related to high nitrogen excretion are problems faced by the poultry industry, and thus efforts have been made to decrease the dietary protein level and consequently nitrogen (N) excretion from broiler production [22]. Furthermore, excessive dietary protein can predispose broiler chickens to NE [23]. On the other hand, reports have shown that supplementation of probiotics in reduced crude protein (RCP) diets can improve bird performance [24, 25] and litter quality and reduced incidence of footpad dermatitis [26, 27]. In this context, the combination of RCP and supplementation of probiotic could potentially contribute to the reduction in NE incidence as well as improve the bird’s welfare.

Previously, performance observations of this study have shown that the supplementation of BA can improve body weight gain (BWG), feed conversion ratio (FCR) and microbial population in broilers [28]. Therefore, it was hypothesized that *Bacillus amyloliquefaciens* CECT 5940 as a probiotic supplement could improve gut environment so as to help reduce adverse effects of NE in chickens and positively affect growth and nutrient uptake in birds fed RCP diets. The current study used two experiments to evaluate the effect of this probiotic on NE challenge and different crude protein levels to test the above hypothesis.

## Methods

All procedures of this study were reviewed and approved by the Animal Ethics Committee of the University of New England (17/127). All procedures involving the birds, including health, care, and use of laboratory animals, were fulfilled within the Code of Practice for the Use of Animals for Scientific Purposes issued by the Australian Code for the Care and Use of Animals for Scientific Purposes (NHMRC, 2013).

### Experimental design and diets

Two experiments were conducted to evaluate the effect of BA supplementation under a necrotic enteritis challenge and/or on different levels of crude protein in diets. All birds in both experiments were as hatched Ross-308 chickens and were obtained from Baiada hatchery in Tamworth, NSW, Australia. Upon arrival, all birds were feather-sexed to enable allocation of the same ratio between males and females in each pen. Both experiments were designed as 2 × 2 factorial arrangement with 480 birds using 32 pens. Each experiment consisted of four treatments, 8 replicates, and 15 birds per pen. In experiment 1, the factors were NE challenge (yes or no) and BA probiotic supplementation (yes or no). In experiment 2, all birds were challenged with NE, and the treatments were dietary CP level (standard or reduced) and BA probiotic supplementation (yes or no).

In both experiments, the dietary concentration of probiotic was 1 × 10^6^ CFU of *Bacillus amyloliquefaciens* CECT 5940 per g of feed. All pens were within the same environmentally controlled facility, equipped with a bell feeder and cup drinkers. Broiler chickens had ad libitum access to feed and water. Ingredients and nutrient composition of the diets are shown in Table 1. The lighting, relative humidity, and temperature were maintained following Ross-308 guidelines [29]. The chickens were fed starter diets from day 0 to 10, and grower diets were fed from day 11 until the birds were sampled at day 16.

**Table 1:** Composition and nutritional content of the experimental diets

Ingredients, %	Starter (d 0–10)		Grower (d 11–16)	
	SCP^c^	RCP^d^	SCP	RCP
Wheat	39.9	49.5	42.6	52.3
Soybean meal	33.2	24.1	29.2	20.0
Sorghum	20.0	20.0	20.0	20.0
Canola oil	2.83	1.38	3.93	2.47
Dicalcium phosphate	1.27	1.33	1.09	1.15
Limestone	1.24	1.26	1.16	1.18
Salt	0.55	0.55	0.55	0.56
*DL*-Methionine	0.34	0.38	0.31	0.35
*L*-Lysine HCl 78%	0.27	0.52	0.25	0.50
*L*-Threonine	0.16	0.27	0.14	0.25
Vitamin premix^a^	0.08	0.08	0.08	0.08
Trace mineral premix^b^	0.10	0.10	0.10	0.10
*L*-Valine	–	0.12	–	0.12
*L*-Isoleucine	–	0.11	–	0.11
*L*-Arginine	–	0.18	–	0.19
Choline chloride 60%	0.03	0.07	0.03	0.07
Phytase	0.01	0.01	0.01	0.01
Titanium dioxide	–	–	0.50	0.50
Nutrients				
CP,%	23.00	21.00	21.5	19.5
AMEn, kcal/kg	3000	3000	3090	3090
Dig. Lys, %	1.20	1.20	1.10	1.10
Dig. Met, %	0.62	0.62	0.57	0.57
Dig. Arg, %	1.33	1.33	1.22	1.22
Dig. Ile, %	0.83	0.83	0.77	0.77
Dig. Val, %	0.90	0.90	0.84	0.84
Choline, mkg/kg	1700	1700	1600	1600
Linoleic acid, %	1.68	1.25	1.99	1.56
Ca, %	0.96	0.96	0.87	0.87
Av. P, %	0.48	0.48	0.44	0.44

^a^Supplied per kilogram of diet: vitamin A, 8255 IU; vitamin D_3_, 3000, IU; vitamin E, 30 IU; vitamin K, 2 mg; thiamine (vitamin B_1_), 4 mg; riboflavin (vitamin B_2_), 6 mg; niacin, 41.2 mg; folic acid, 1 mg; biotin, 0.25 mg; pyridoxine, 4 mg; choline, 1301 mg; pantothenic acid, 11 mg; vitamin B_12_, 0.013 mg. ^b^Supplied per kilogram of diet: Cu (sulphate), 16 mg; Fe (sulphate), 40 mg; I (iodide), 1.25 mg; Se (selenate), 0.3 mg; Mn (sulphate and oxide), 120 mg; Zn (sulphate and oxide), 100 mg; cereal-based carrier, 128 mg; mineral oil, 3.75 mg., ^c^Standard crude protein. ^d^Reduced crude protein

### Necrotic enteritis challenge

To induce subclinical NE infection in birds, the University of New England NE challenge procedure as described by Rodgers et al. [30] was followed. In brief, on day 9 all challenged birds were orally inoculated with 1 mL/bird field *Eimeria* strains (*E. acervulina* at 5000 oocytes/mL, *E. maxima* at 5000 oocytes/mL, *E. brunetti* at 2500 oocytes/mL); chickens in the non-challenged groups were inoculated with 1 mL of sterile PBS. Primary poultry isolates of *C. perfringens* (EHE-NE18) were obtained from CSIRO Livestock Industries, Geelong, Australia and was incubated overnight at 39 °C in 100 mL of sterile thioglycollate broth (USP alternative; Oxoid) followed by subsequent overnight incubation of 1 mL of the previous culture in 100 mL of cooked meat medium (Oxoid), and then in 700 mL of thioglycollate broth (USP alternative; Oxoid) containing starch (10 g/L) and pancreatic digest of casein (5 g/L) to obtain the challenge inoculum. After preparation of the inoculums, 1 mL of fresh inoculums containing approximately 10^8^ CFU/mL *C. perfringens* was inoculated to the chickens on day 14 and 15. Chickens in the unchallenged groups were gavaged with sterile thioglycolate medium as a sham treatment.

### Blood and intestinal tissue collection

In both experiments, at day 16, two male birds from each pen were randomly chosen and gavaged with 1 mL fluorescein isothiocyanate-dextran (FITC-d) (at a dose of 4.17 mg/kg bird average body weight; molecular weight 4000, Sigma–Aldrich Co., Sydney, Australia), 2.5 h prior to euthanization [31]. Birds were electrically stunned, and blood was collected from the jugular vein in clot activator vacutainer tubes, immediately followed by decapitation and dissection of birds. Approximately 2 cm of the proximal jejunum tissue was excised, flushed with chilled sterile PBS before they were collected in 2 mL Eppendorf tubes containing 1.5 mL RNA later (Qiagen, Hilden, Germany) and stored at − 20 °C until the extraction of RNA. Blood samples were kept in room temperature for approximately 3 h followed by 15 min centrifugation at 1000×*g* to separate red blood cells from serum. Fluorescent levels in serum samples were measured with an excitation wavelength of 485 nm and an emission wavelength of 528 nm on a Synergy HT, Multi-mode microplate reader SpectraMax M2e (Molecular Devices, San Jose, USA).

### RNA extraction and cDNA synthesis

For each sample, total RNA from each jejunal tissue was extracted after homogenization in TRIsureTM (Bioline, Sydney, Australia) following the manufacturer’s instructions. All RNA samples were purified with the RNeasy Mini Kit, (Qiagen, Hilden, Germany) following the manufacturer instructions. The quantity and purity of the samples were measured with NanoDrop ND-8000 spectrophotometer (Thermo Fisher Scientific, Waltham, USA), and the RNA Nano 6000 kit was used to measure RNA integrity on the Agilent 2100 Bioanalyzer (Agilent Technologies, Inc., Waldron, Germany). The samples were considered as high-quality if the value of 260/230 was > 2.0, 260/280 value between 2.0–2.2, and the RIN number was higher than 7. The extracted RNA of each sample was reverse transcribed with the QuantiTect Reverse Transcription Kit (Qiagen, Hilden, Germany) according to the manufacturer instructions. Rotorgene 6000 Real-Time PCR machine (Corbett, Sydney, Australia) was employed to convert RNA to cDNA. The cDNA samples were diluted 10 times with nuclease-free water and stored at − 20 °C until required.

### Real-time quantitative polymerase chain reaction (RT-qPCR)

Amplification and detection were carried out in duplicates using an SYBR Green kit SensiFAST SYBR No-ROX (Bioline, Sydney, Australia) with Rotorgene 6000 real-time PCR machine (Corbett Research, Sydney, Australia). The PCR reaction was performed in a volume of 10 μL containing 5 μL of 2× SensiFAST, 400 mmol/L of each primer and 2 μL of 10× diluted cDNA template. For choosing two suitable reference genes for the analysis, the stability of eight widely used house-keeping genes (TATA-Box Binding Protein (*TBP*), glyceraldehyde-3-Phosphate Dehydrogenase (*GADPH*), hydroxymethylbilane synthase (*HMBS*), tyrosine 3-monooxygenase/tryptophan 5-monooxygenase activation protein zeta (*YWHAZ*), cystatin C (*CST3*), hypoxanthine phosphoribosyltransferase 1 (*HPRT1*), carbonic anhydrase 2 (*CA2*) and Beta 2-microglobulin (*B2M*) were checked in the geNorm module in qbase+ software version 3.0 (Biogazelle, Belgium) to calculate the gene expression stability measure (geNorm M) [32]. The two most stable genes with lowest M value (< 0.5) were chosen as suitable reference gene candidates (*HMBS* and *YWHAZ*). Next, all raw Cq values for all candidate target genes were imported into qbase + version 3.0 (Biogazelle, Belgium) and analysed against the two reference genes *HMBS* and *YWHAZ*. The qbase + applies an arithmetic mean method to transform logarithmic Cq values to linear relative quantity using exponential function for relative quantification of genes and the arithmetic mean of average Cq is scaled to a given target sample [32]. The result table shows normalized relative quantities (NRQ) values that are calculated across all unknown samples per target gene. Primers used in this study were either sourced from literature or designed using NCBI Primer-BLAST tool (https://www.ncbi.nlm.nih.gov/tools/primer-blast/) as shown in Table 2. All qPCR primers were checked for specificity using Agilent DNA 1000 Kit with the Agilent 2100 Bioanalyzer (Agilent Technologies, Inc., Waldron, Germany).

**Table 2:** Sequences of primers used for quantitative real-time PCR

Gene	Accession No	Sequence (5'→3')	Size, bp	Annealing T, °C	Reference
*CASP3*	NM_204725.1	F-TGGTGGAGGTGGAGGAGC R- GTTTCTCTGTATCTTGAAGCACCA	110	62	This study
*CASP8*	NM_204592.2	F-GGAGCTGCTATCGGATCAAT R-GGAGCTGCTCTATCGGATCAAT	126	60	This study
*IgA*	S40610.1	F- GTCACCGTCACCTGGACACCA R- ACCGATGGTCTCCTTCACATC	192	64	[33]
*IgG*	X07174.1	F- ATCACGTCAAGGGATGCCCG R- ACCAGGCACCTCAGTTTGG	118	60	[34]
*IgM*	X01613.1	F- GCATCAGCGTCACCGAAAGC R- TCCGCACTCCATCCTCTTGC	98	60	[34]
*MUC2*	XM 001234581.3	F- CCCTGGAAGTAGAGGTGACTG R- TGACAAGCCATTGAAGGACA	143	60	[35]
*OCLD*	NM_205128.1	F- ACGGCAGCACCTACCTCAA R- GGGCGAAGAAGCAGATGAG	123	60	[36]
*TJP1*	XM_413773.4	F- GGATGTTTATTTGGGCGGC R- GTCACCGTGTGTTGTTCCCAT	187	60	[37]
*CLDN1*	NM_001013611.2	F- CTTCATCATTGCAGGTCTGTCAG R- AAATCTGGTGTTAACGGGTGTG	103	60	[37]
*IL18*	NM_204608.1	F- TGTGTGTGCAGTACGGCTTAG R- CTTACAAAAGGCATCGCATTC	79	60	[38]
*Il-2*	NM_204153.1	F- TCTGGGACCACTGTATGCTCT R- ACACCAGTGGGAAACAGTATCA	256	60	[39]
*IFN-y*	Y07922	F- AGCTGACGGTGGTGGACCTATTATT R- GGCTTTGCGCTGGATTC	259	60	[40]
*ATP1A1*	NM_205521.1	F- GTCAACCCGAGGGATGCTAA R- ACTGCTACAATGGCACCCTG	179	60	[41]
*B*^*0*^*AT*	XM_419056.5	F- GTGTTTGGAACCCTAAATACGAGG R- TAGCATAGACCCAGCCAGGA	72	60	[41]
*GLUT2*	NM_207178.1	F- TGATCGTGGCACTGATGGTT R- CCACCAGGAAGACGGAGATA	171	60	[41]
*SI*	XM_015291762.1	F- GCTTTAAGATGGGCAAGAGGAAG R- CCACCACCAGGCAAAAGAGG	65	60	[41]
*HMBS*	XM 417846.2	F- GGCTGGGAGAATCGCATAGG R- TCCTGCAGGGCAGATACCAT	131	60	[42]
*YWHAZ*	NM_001031343.1	F- TTGCTGCTGGAGATGACAAG R- CTTCTTGATACGCCTGTTG	61	60	[43]

### Statistical analysis

The data for each experiment was analyzed by a 2-way ANOVA using the General Linear Models (GLM) procedure of SPSS statistics version 22 (IBM Corporation) for the main effect and interactions of NE infection and BA supplementation in experiment 1 and crude protein level and BA supplementation in experiment 2. If a significant effect was detected, differences between treatments were separated by a least significant difference test (Tukey’s test). Differences between mean values were considered significant at *P* ≤ 0.05.

## Results

### Gut integrity

**Fig. 1:**
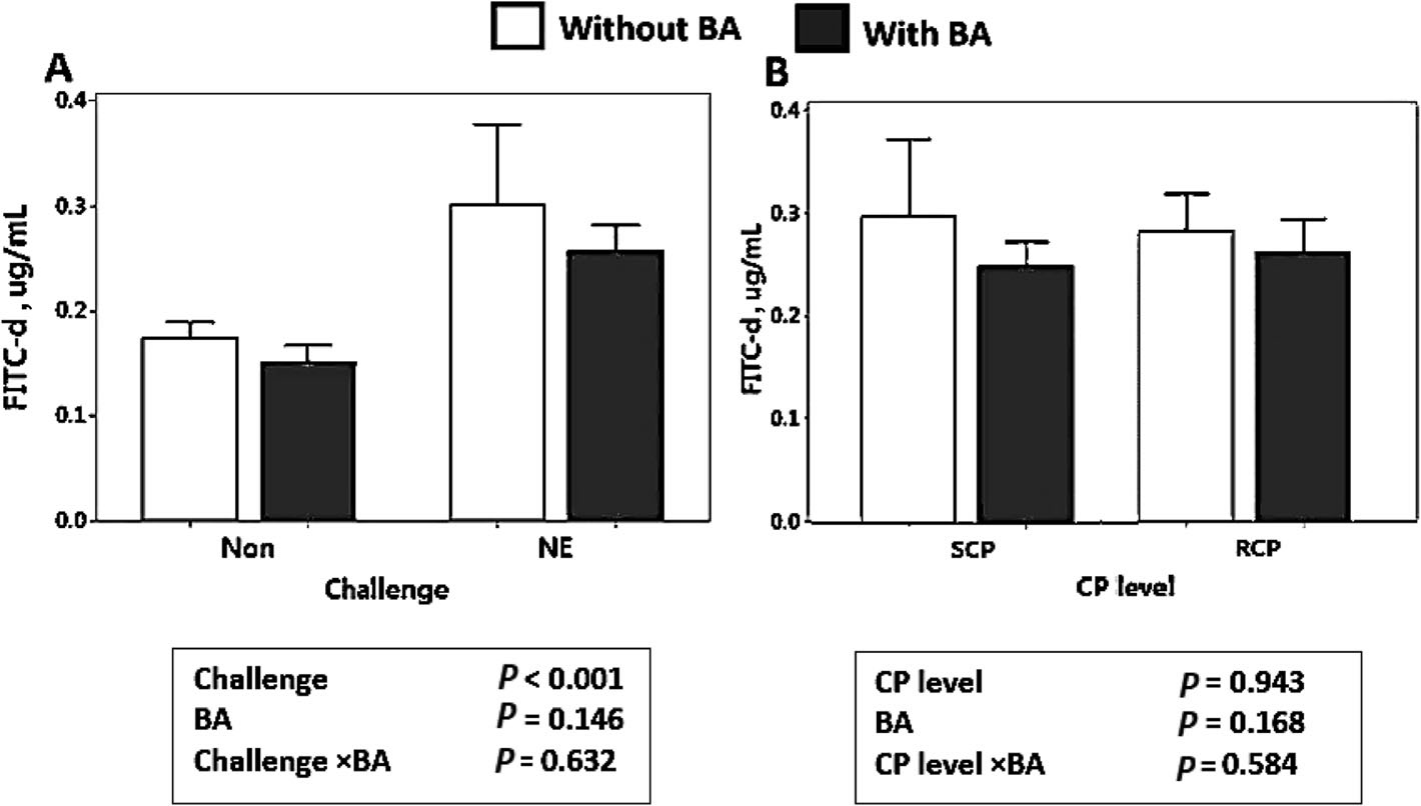
Effect of *Bacillus amyloliquefaciens* CECT 5490 (1.0 × 10^6^ CFU/g of feed) (BA) on fluorescein isothiocyanate-dextran (FITC-d) concentration in broiler serum at day 16, (**a**) Effect of BA supplementation and NE challenge on broiler serum FITC-d concentration; (**b**) Effect of crude protein levels and BA supplementation on broiler serum FITC-d concentration. CP: Crude protein. SCP: Standard protein diets, RCP: Reduced protein diets (2% lower than standard diets). Error bars show SEM for 8 replicates for each sample test

As illustrated in Fig. 1a, gut integrity analysis showed higher serum FITC-d concentrations (*P* < 0.001) in birds challenged with NE compared to non-challenged birds in experiment 1. Additionally, supplementation of BA did not significantly alter gut integrity (*P* > 0.05) in this experiment. In experiment 2, crude protein levels and BA supplementation (Fig.1b) show no significant effect on FITC-d concentrations in chickens’ serum (*P* > 0.05).

### Downregulation of *CASP3* and *CASP8* by BA supplementation

**Fig. 2:**
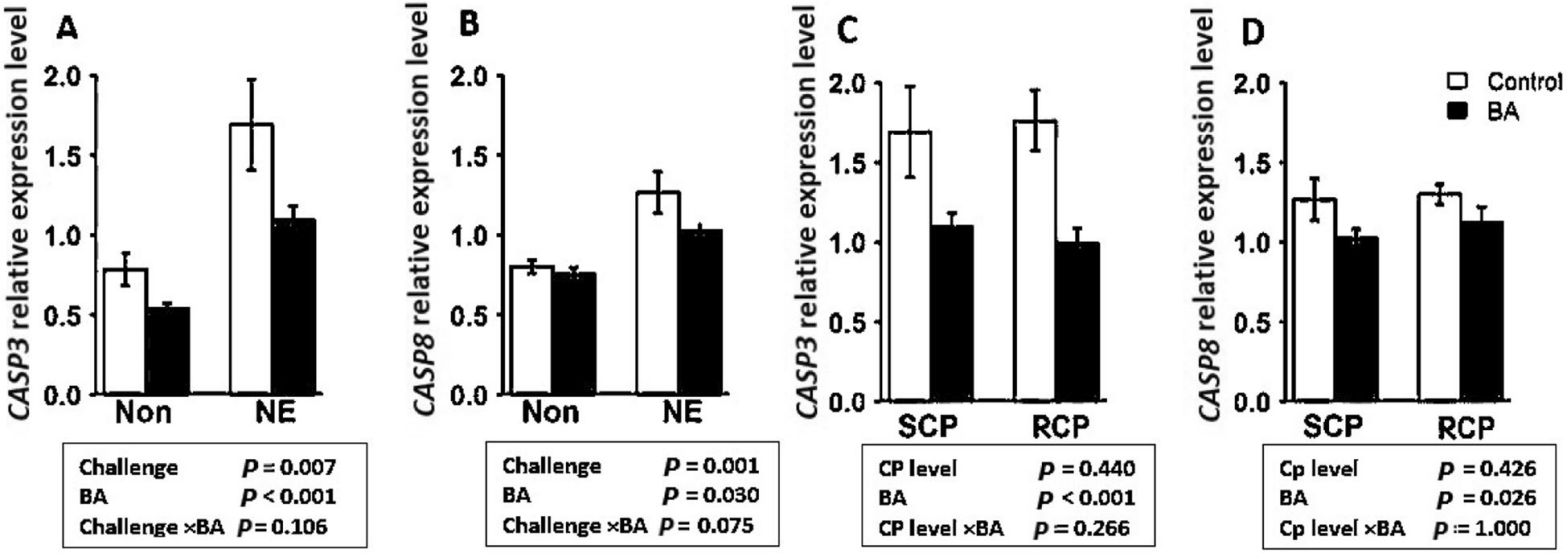
Effect of *Bacillus amyloliquefaciens* CECT 5490 (1.0 × 10^6^ CFU/g of feed) (BA) on two apoptosis related genes at day 16. (**a-b)** Effect of NE challenge and BA supplementation on caspase-3 (*CASP3*) and caspase-8 (*CASP8*) expression; (**c-d**) Effect of crude protein (CP) level and BA supplementation on *CASP3* and *CASP8* expression. Non: non-challenged; NE: NE challenged; SCP: Standard protein diet; RCP: Reduced protein diet RCP: Reduced protein diets (2% lower than standard diets). Genes used as reference genes were *HMBS* and *YWHAZ*. Error bars show SEM for 8 replicates for each treatment

The mRNA expression of two apoptotic genes in both experiments are shown in Fig. 2. In experiment 1, challenged birds had a significantly higher expression of caspase-3 (*CASP3*) (*P* < 0.01) and caspase-8 (*CASP8*) (*P* < 0.001) compared to the unchallenged groups. Furthermore, BA supplementation decreased the mRNA expression of *CASP3* (*P* < 0.001) and *CASP8* (*P* < 0.05) in both challenged and unchallenged birds (Fig. 2a and b). In experiment 2, the addition of BA in diets downregulated both *CASP3* (*P* < 0.001) and *CASP8* (*P* < 0.05) in all birds regardless of the protein level of the diet (Fig. 2c and d). Crude protein level did not have a significant effect on the expression level of *CASP3* and *CASP8* (*P* > 0.05).

### Expression of genes encoding digestive enzymes and nutrient transporters

**Fig. 3:**
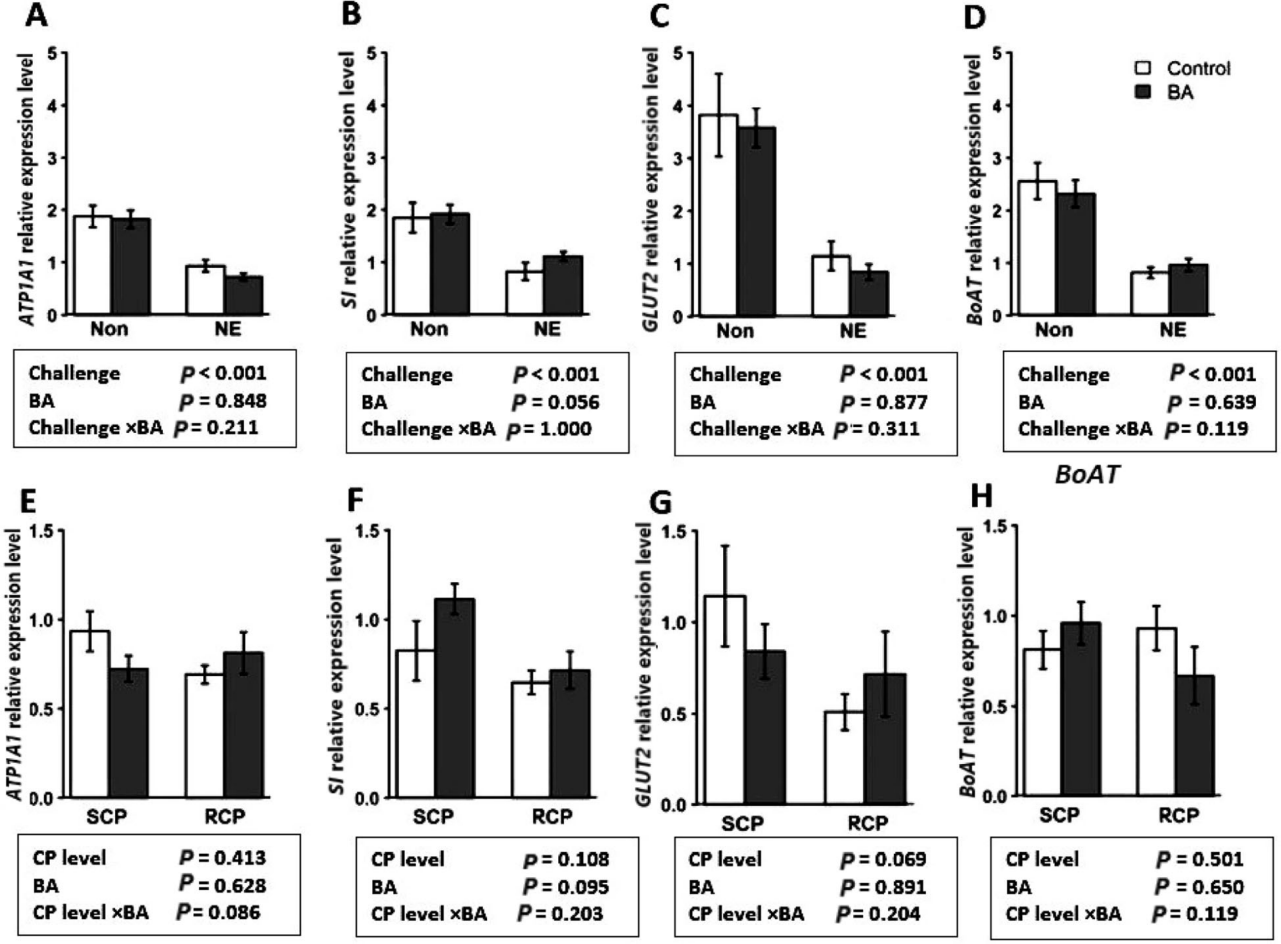
Effect of *Bacillus amyloliquefaciens* CECT 5490 (1.0 × 10^6^ CFU/g of feed) (BA) on genes related to enzyme and nutrient transporters at day 16. (**a-d)** Effect of NE challenge and BA supplementation on the expression of two enzyme (ATPase Na^+^/K^+^ transporting subunit alpha-1 (*ATP1A1*) and sucrase-isomaltase (*SI*) and nutrient transporter genes (glucose transporter-2 (*GLUT2*) and neutral aminoacid transporter (*B*^*0*^*AT*) (**e**-**d**) Effect of crude protein (CP) level and BA supplementation on the expression of two enzyme (*ATP1A1* and *SI*) and nutrient transporter genes (*GLUT2* and *B*^*0*^*AT*). Non: non-challenged; NE: NE challenged; SCP: Standard protein diet; RCP: Reduced protein diet (2% lower than standard diets). Genes used as reference genes were *HMBS* and *YWHAZ*. Error bars show SEM for 8 replicates for each treatment

Expression of genes related to nutrient transporters and digestive enzymes were evaluated in the jejunum tissue of birds in both experiments. The effect of NE challenge and BA supplementation is shown in Fig. 3a-d. The expression of *GLUT2*, *B*^*0*^*AT*, *ATP1A1*, and *SI* were downregulated by NE challenge (*P* < 0.001), and BA did not significantly alter the expression of these genes. Results from experiment 2 (Fig. 3e-h) showed that BA supplementation and crude protein levels did not significantly affect the expression of *GLUT2*, *B*^*0*^*AT*, *ATP1A1*, and *SI* genes (*P* > 0.05).

### Upregulation of *OCLD* and *MUC2* by BA supplementation

**Fig. 4:**
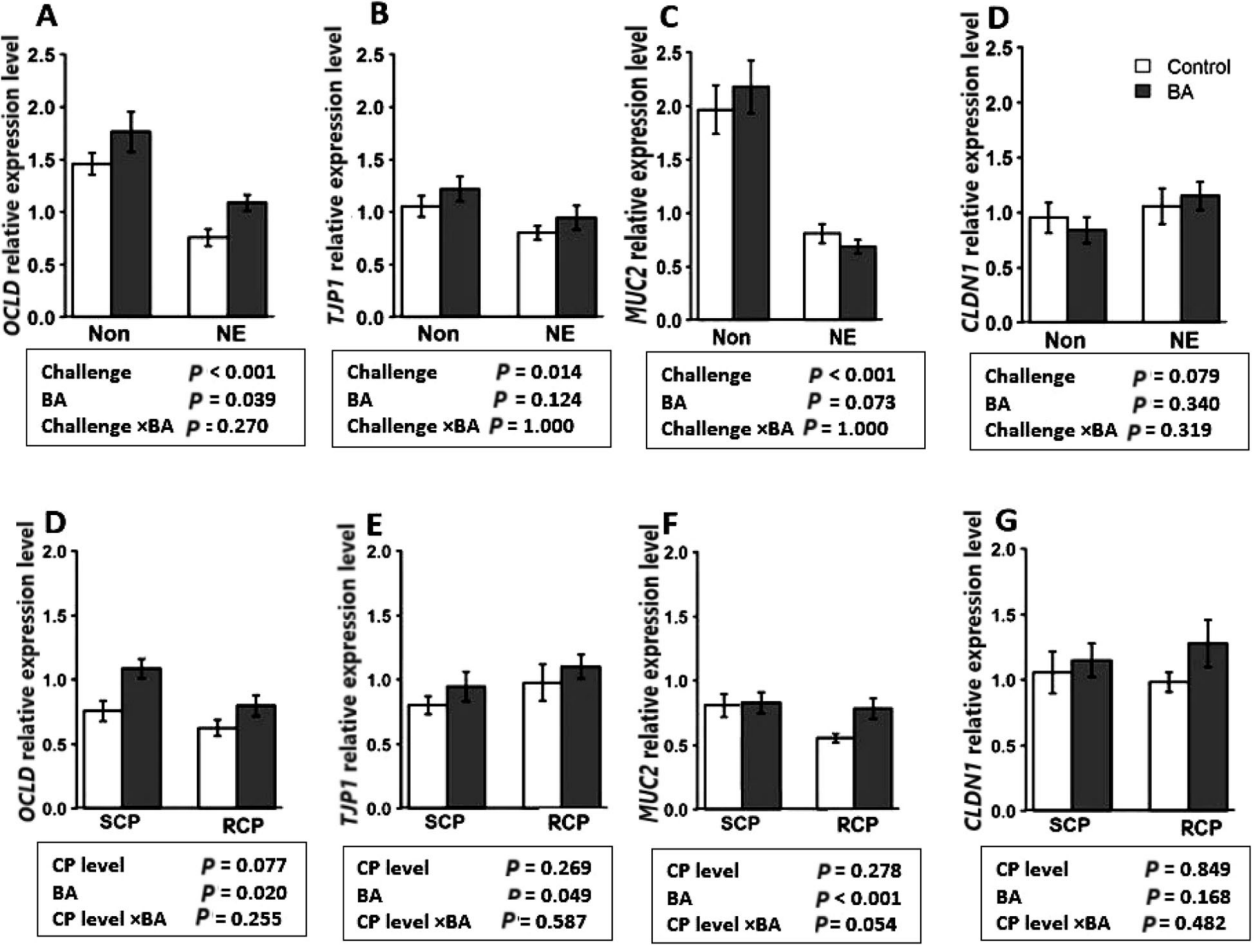
Effect of *Bacillus amyloliquefaciens* CECT 5490 (1.0 × 10^6^ CFU/g of feed) (BA) on intestinal tight junction related gene expression in broilers at day 16. (**a-d**) Effect of NE challenge and BA supplementation on occludin (*OCLD*), tight junction proteins-1 (*TJP1*), mucin-2 (*MUC2*), and claudin-1 (*CLDN1*) expression level; (**e**-**d**) Effect of crude protein (CP) level and BA supplementation on *OCLD*, *TJP1*, *MUC2*, and *CLDN1* expression level. Non: non-challenged; NE: NE challenged; SCP: Standard protein diet; RCP: Reduced protein diet (2% lower than standard diets). Genes used as reference genes were *HMBS* and *YWHAZ*. Error bars show SEM for 8 replicates for each treatment

The expression of tight junction proteins and *MUC2* in different groups is shown in Fig. 4. The expression of occludin (*OCLD)* (*P* < 0.001), *TJP1* (*P* < 0.05) and *MUC2* (*P* < 0.001), were downregulated by the NE challenge (Fig. 4a-c). The supplementation of BA significantly increased the expression level of *OCLD* (*P* < 0.05) regardless of the challenged. In experiment 2, BA supplementation increased *OCLD* expression (*P* < 0.05), *TJP1* (*P* < 0.05) and *MUC2* (*P* < 0.001) regardless of the crude protein level in diets (Fig. 4d-f). The expression of *CLDN1* was not affected in either of the experiments (*P* > 0.05).

### Upregulation of *IgA*, *IgM*, and downregulation of *INF-γ* by BA supplementation

**Fig. 5:**
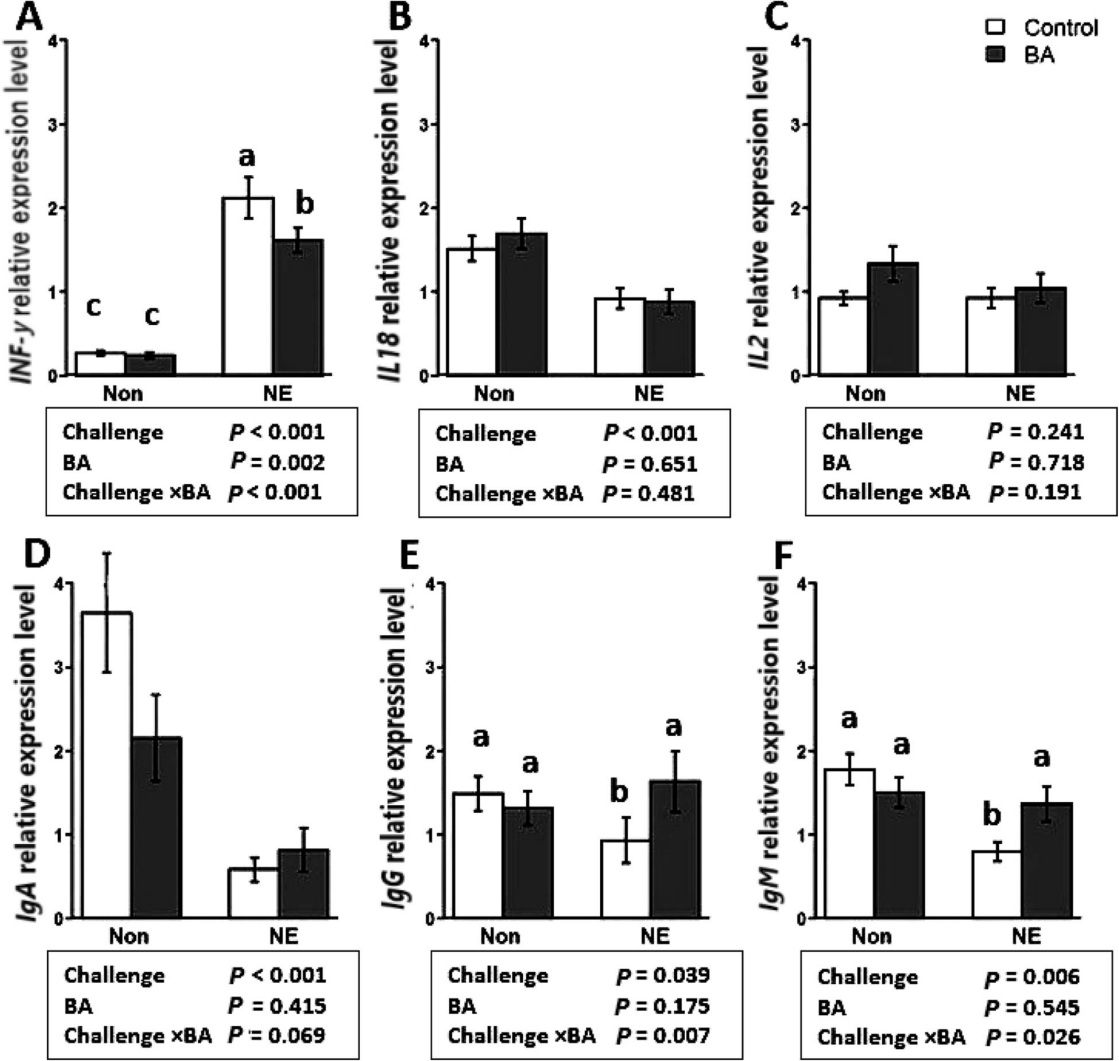
Effect of *Bacillus amyloliquefaciens* CECT 5490 (1.0 × 10^6^ CFU/g of feed) (BA) on the expression of intestinal immunity related genes in broilers at day 16. Non: non-challenged; NE: NE challenged. *IFN-γ*: interferon-γ; *IL18*: Interleukin-18; *IL2*: Interlukin-2; *IgA*, *IgG* and *IgM*: Immunoglobulin A, G and M. ^a-c^ bars with different letters significantly differ on the basis on Tukeys’ multiple tests (*P* < 0.05). Genes used as reference genes were *HMBS* and *YWHAZ*. Error bars show SEM for 8 replicates for each treatment

**Fig. 6:**
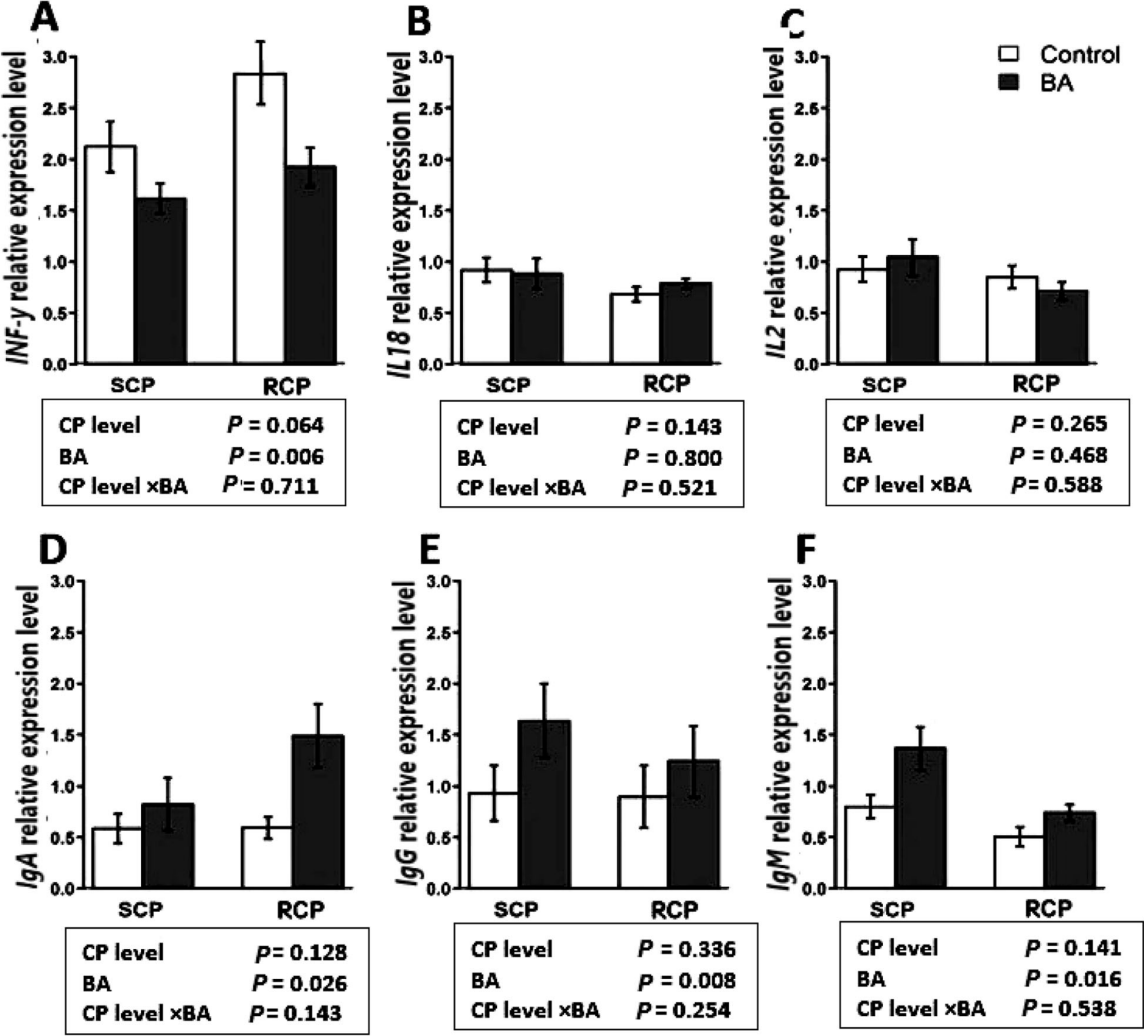
Effect of *Bacillus amyloliquefaciens* CECT 5490 (1.0 × 10^6^ CFU/g of feed) (BA) on the expression of intestinal immunity related genes in broilers at day 16. SCP: Standard protein diet; RCP: Reduced protein diet. *IFN-γ*: interferon-γ. *IL18*: Interleukin-18; *IL2*: Interlukin-2; *IgA*, *IgG* and *IgM*: Immunoglobulin A, G and M. SCP: Standard protein diet; RCP: Reduced protein diet (2% lower than standard diets). Genes used as reference genes were *HMBS* and *YWHAZ*. Error bars show SEM for 8 replicates for each treatment

Figure 5 illustrates the mRNA expression of six immunity-related genes affected by NE challenge and BA supplementation. As shown in Fig. 5a, an NE challenge × BA interaction was observed for interferon-γ (*IFN-γ*) where BA supplementation downregulated *IFN-γ* expression only in the NE challenged birds (*P* < 0.001). An NE challenge × BA interaction was observed for *IgG* (*P* < 0.01) and *IgM* (*P* < 0.05), where the NE challenge with no BA supplementation shows the lowest values, however, the supplementation of BA give similar results as the unchallenged birds with or without BA supplementation. The effect of crude protein level and BA is illustrated in Fig. 6. Birds fed with BA showed downregulation in the expression of *INF-γ* (*P* < 0.01) and upregulation of *IgA* (*P* < 0.05), *IgG* (*P* < 0.01), and *IgM* (*P* < 0.05), regardless of CP level in the diet. No difference was observed for the expression of *IL18* (*P* > 0.05) in response to the BA supplementation and CP level in diet as main effects or interactively.

## Discussion

This study investigated the expression of a series of genes encoding proteins related to immunity, intestinal integrity, apoptosis and mucin production in broiler jejunum tissue in response to the supplementation of BA-5460 when fed diet with different levels of CP and/or challenged with NE. The BA supplementation reduced the apoptosis gene expression and had a positive effect on the immunity and mucosal gene expression in the intestine. The present data lead to the acceptance of our hypothesis that BA, as a probiotic, can improve gut health of broiler chickens likely through the modulation of intestinal genes responsible for a variety of functions in the intestine.

We have previously reported that successful subclinical NE challenge was applied in this experiment, as performance traits were significantly affected by the challenge [28]. The NE challenge significantly impaired BWG and FCR in birds, and increased lesion scores in these groups [28]. Most of the genes evaluated in this experiment were affected by the NE challenge. The higher expression of caspase and lower expression of immunoglobulin and interleukin proteins prove the altered cell death and impaired immunity system in the challenged birds that further confirm the occurrence of the NE disease in the study.

The small intestine has a primitive function in the digestion and nutrient absorption processes. The structure and function of the intestinal mucosa, which in part is associated with the morphological characteristics, highly depends on the balance between proliferation and apoptosis [44]. In the present study, lower expression of *MUC2* in the NE challenged groups can be related to the inflammation in the gut. Inflammatory lesions can reduce goblet cells, which produce mucin and contain the *MUC2* gene, prevent the mucosal layer from replenishing and increase the chance for further infection, bacterial translocation and inflammation in the intestine [38, 45]. In this experiment, the expression of *MUC2* in the birds fed with BA was higher compared to the non-supplemented groups indicating positive effects of this probiotic on the protection of the birds from disease. Previous studies have similarly reported the positive effects of *Bacillus*-based probiotics on the *MUC2* mRNA expression in chicken jejunum [46, 47].

Activation of pro-inflammatory cytokines such as IFN-γ and/or tumor necrosis factor-α (TNF-α) can promote cell death by apoptosis and also lead to β-cell destruction [48]. Our results demonstrate that the addition of BA has downregulated expression of both pro-inflammatory cytokines (*IFN-γ*) and apoptosis genes (*CASP3* and *CASP8*) in NE challenged birds. Caspases are pre-apoptotic proteins, expressed in most of the cells that are active in the cell death pathways. The *C. perfringens* enterotoxin (CPE) can bind to enterocytes then oligomerize into a hexametric prepore on the cell membrane surface and form an active pore, which can then cause an increased influx of Ca^2+^ that may lead to enterocyte cell death [49]. The CASP3 and CASP8 proteins are executioner caspases which can be used as markers for cell apoptosis [50]. The results from the current study revealed that the supplementation of BA reduced the relative expression of both *CASP3* and *CASP8* in the challenged birds, as has also been shown with *Bacillus* spp. previously [51]. The BA supplementation has shown to reduce CASP3 activity and reduce the activation of other apoptosis pathways related to oxidative stress in porcine epithelium cell line [52, 53]. Lower activity of cell death pathways implies a lower inflammatory pathway activity, which may lead to improved health of the intestine.

The intestinal epithelium is in permanent contact with dynamic enteric flora. The intestinal barrier and tight junctions are major defense mechanisms used to maintain epithelial integrity for protecting the organism from the environment. In the present study, a higher level of FITC-d concentrations in the bloodstream and lower mRNA expression of tight junction genes such as *OCLD* and *TJP1* in the NE infected birds indicate the damage in the paracellular permeability, which has been observed in previous studies [54, 55]. The addition of BA upregulated the expression of *OCLD* in challenged birds. The expression level of this protein is known to correlate with the number of tight junction strands in the epithelia [56]. Tight junctions regulate nutrient absorption, homeostasis, and defense against invading pathogens in the intestine and are known to be the main indicators for intestinal epithelium health. *Bacillus subtilis* as a supplemented probiotic in mice with inflammatory bowel disease has shown to upregulate expression of tight junction genes, including *OCLD*, and thus improve the barrier function [57]. A mixture of *B. subtilis* and *Saccharomyces boulardii* as probiotics have shown to increase the expression of tight junction genes such as *OCLD*, claudin-2 (*CLDN2*), and claudin-2 (*CLDN3*) in broilers [58].

Furthermore, a link between increased levels of pro-inflammatory cytokines and intestinal permeability has been reported [59]. Secretion of inflammatory cytokines that are immunoregulatory peptides can alter tight junction gene expression [60]. Interferon-γ elaborates regulation of inflammatory immune responses, and *C. perfringens* infection has shown to increase the expression of this gene [55, 61]. This protein can rearrange and redistribute the actin cytoskeleton in the intestine and increase paracellular permeability [62]. It can also regulate intestinal epithelial cell proliferation and apoptosis through AKT-β-catenin pathways [63]. *Bacillus*-based probiotics have shown to promote the synthesis of endogenous antimicrobial peptides in the gut, thus enhance the innate immune function [64]. Studies have shown that these probiotics can reduce the mRNA expression of *IFN-γ*, which may be attributed to decreased pathogen load in the gut [61]. The improvement in tight junction protein expressions mostly observed in experiment 2 could be related to the downregulated expression of *IFN-γ* triggered by BA. The BA supplementation can alleviate lipopolysaccharide-induced intestinal damage in chicken [65] and reduce mRNA levels of pro-inflammatory cytokines such as tumor necrosis factor in mice [66]. The suppression of inflammatory cytokines by the supplementation of BA may have led to the improved intestinal gut integrity.

The mucosal plasma cells in the lamina propria, produce secretory immunoglobulins such as IgA, and IgM that act as the first line of defense in the small intestine and other luminal surfaces [67]. All NE challenged birds fed with BA supplemented diets showed higher expression of immunoglobulins in the intestine. One reason for the lower expression of *IgA* and *IgM* observed in the challenged birds of this study, could be due to the damaged intestinal structure, along with the downregulation of the genes involved in the amino acid uptake in the intestinal tissue [68–70]. Immunoglobulin proteins such as IgA, IgM, and IgG, are present in the enterocyte brush border and are delivered to the mucus layer [16]. Similar to the current results, Wang et al. [71] reported a decrease of IgA+ B cells in NE infected chickens. *Bacillus*-based probiotics have shown to increase the availability of some amino acids [25] and to increase digestive enzyme activity, protein efficiency, and nutrient retention in broilers [72]. There is a direct correlation between amino acid balance on mucin production [73], thus the improved amino acid uptake could have led to increased expression of *MUC2* observed in the current results. We have previously observed the positive effects of BA supplementation on amino acid digestibility [28]. It should be noted that the number of genes evaluated in this study are extremely limited. There are immense numbers of nutrient transporter, and enzyme proteins that may have been affected by BA. Further study is needed to clearly understand the effects of BA supplementation on gut health and nutrient uptake.

## Conclusion

Taken together, the results of this study suggest that the dietary supplementation of BA could alleviate the undesirable effects of NE of intestinal cell death and immune responses, hence further improve performance. Further investigation on the effects of this *Bacillus* strain is needed for a better understanding of the mechanisms underlying possible positive effects of the probiotic supplementation to the birds under challenge and unchallenged conditions.

## Data Availability

Data may be provided following request to the corresponding author.

## Abbreviations

NE: Necrotic enteritis
BA: *Bacillus amyloliquefaciens* CECT 5940
SCP: Standard protein diets
RCP: Reduced protein diets
FITC-d: Fluorescein isothiocyanate-dextran
CASP3: Caspase-3
CASP8: Caspase-8
CLDN1: Claudin 1
OCLD: Occludin
MUC2: Mucin 2
IgA: Immunoglobulin-A
IgM: Immunoglobulin-M
IgG: Immunoglobulin-G
TJP1: Tight junction protein-1
GLUT2: Glucose transporter-2
ATP1A1: ATPase Na^+^/K^+^ Transporting subunit alpha-1
SI: Sucrase-isomerase
B^0^AT: Neutral amino acid transporter
IL18: Interleukin-18
IL-2: Interleukin-2
IFN-γ: Interferon-γ
